# Hemosiderin in sputum macrophages may predict infective exacerbations of chronic obstructive pulmonary disease: a retrospective observational study

**DOI:** 10.1186/s12890-017-0408-4

**Published:** 2017-04-12

**Authors:** Sindu Mohan, Terence Ho, Melanie Kjarsgaard, Katherine Radford, A. S. M. Borhan, Lehana Thabane, Parameswaran Nair

**Affiliations:** 1 Division of Respirology, St Joseph’s Healthcare and Department of Medicine, McMaster University, Hamilton, ON Canada; 2 Department of Health Research Methods, McMaster University, Hamilton, ON Canada; 3 Firestone Institute for Respiratory Health, St Joseph’s Healthcare, 50 Charlton Avenue East, Hamilton, ON L8N 4A6 Canada

**Keywords:** COPD, Exacerbations, Sputum, Macrophage, Hemosiderin

## Abstract

**Background:**

Infective exacerbations of COPD are common and are accompanied by neutrophilic bronchitis in sputum. Increased respiratory iron content has been associated with respiratory tract infection, though it is unclear if this represents a predisposing factor for infection or the sequelae of inflammation. Iron overload, as assessed in the airways, may be an important biomarker for recurrent infective exacerbations of COPD. The purpose of our study was to determine if hemosiderin in sputum macrophages is related to infective exacerbations of COPD.

**Methods:**

We undertook a retrospective observational study of 54 consecutive patients who presented with an exacerbation of COPD and had sputum examined including assessment for hemosiderin in alveolar macrophages. The relation between infective exacerbations in the previous two years and the percent of hemosiderin-positive macrophages was analyzed with linear regression. To account for the non-parametric distribution of infective exacerbations, negative binomial regression modelling was used to account for other covariates.

**Results:**

The percent of hemosiderin positive alveolar macrophages (hemosiderin index), analyzed parametrically and non-parametrically, demonstrated a significant correlation with increasing numbers of infective exacerbations in the previous two years. In a multivariate regression analysis, hemosiderin index was an independent predictor of infective exacerbations. COPD patients with raised hemosiderin index (≥20%) had higher levels of sputum IL-6 compared to patients with lower levels (<20%).

**Conclusions:**

High hemosiderin index in sputum alveolar macrophages measured at the time of AECOPD may be related to the frequency of infective exacerbations of COPD.

## Background

Chronic obstructive pulmonary disease (COPD) is predicted to be the third leading cause of mortality according to new estimates by the World Health Organization [1]. Exacerbations are considered key events in the natural history of this condition, as they are characterized by episodic decline in respiratory health from a stable state. COPD exacerbations are associated with a number of negative health outcomes, and also present a major economic burden [2, 3]. Preventing exacerbations is therefore a crucial part of COPD management.

The etiology of acute exacerbations of COPD (AECOPD) includes infectious as well as non-infectious causes. Quantitative cell counts in sputum have been useful in characterizing patients with COPD, with eosinophils in sputum indicating a steroid-responsive bronchitis and intense neutrophilia generally indicating an infective bronchitis that would respond to antibiotics [4]. There is a strong correlation between frequency of exacerbations and mortality that is independent of disease severity [3, 4]. Phenotyping patients with frequent exacerbations is therefore a focus of COPD research, with the aim of developing targeted exacerbation prevention strategies [3]. The strongest determinant of future exacerbation is a prior history of exacerbations, though a variety of biomarkers that may help predict the frequent-exacerbation phenotype have been reported [5, 6]. Several gene variations have been implicated in predisposing to frequent exacerbations, many of which are related to iron metabolism [7–9].

Iron overload may play a role in respiratory infection, as nearly all pathogens require a constant supply of iron to sustain an infection, and microbial virulence increases with their ability to obtain growth essential iron from the host [10]. Several studies have shown an association between iron and respiratory infections, with occupational exposures to iron-contaminated dust and fumes resulting in higher rates of pneumonia, influenza and bronchitis [11–13]. Iron is of fundamental importance in mediating oxidative lung injury and the pathogenesis of COPD by generating toxic hydroxyl radicals and promoting intracellular bacterial growth [14, 15]. The vast majority of iron in the respiratory tract, including excessively accumulated iron, is stored within alveolar macrophages (AMs) and airway epithelial cells in an insoluble complex of hemosiderin to limit oxidative damage [16]. We had previously demonstrated that hemosiderin in macrophages can be reliably identified by Perl’s Prussian Blue staining in sputum in patients with COPD [17]. Tobacco smoke is one potential source of iron accumulation in the lower respiratory tract due to its inherently high iron content and by promoting the activity of iron chelators, known as siderophores [16]. Other proposed mechanisms of AM hemosiderin accumulation in COPD include pulmonary micro-hemorrhage, infection, genetic predisposition, elevated left ventricular end-diastolic pressure (LVEDP), and chronic inflammatory states [17, 18]. Though the exact longevity of hemosiderin in AMs is uncertain, AMs are known to have a slow turnover rate, and their status may be reflective of previous insults [19].

Iron availability is primarily controlled by hepcidin, which causes hypoferremia through the degradation of the iron efflux protein, ferroportin [20]. In response to inflammatory stimuli, Interleukin-6 (IL-6) triggers hepcidin release, which sequesters iron in the intracellular compartment and limits microbial access to free iron. IL-6 can be measured in the serum and sputum, with the sputum levels thought to be more representative of the local inflammatory response [21]. States of chronic inflammation can lead to excess hepcidin production, which ultimately may lead to iron overload [20]. As COPD is associated with infection and inflammation, respiratory iron content may be elevated, which could plausibly predispose to frequent infective AECOPD.

The objective of this study was to determine if the percent of hemosiderin positive sputum macrophages measured at the time of AECOPD is a predictor of the frequency of infective exacerbations in COPD. The secondary objective was to explore the potential role of chronic inflammation in respiratory tract iron metabolism, by examining the association between IL-6 levels in sputum and hemosiderin-laden AMs.

## Methods

### Patients

We undertook a retrospective study of 54 consecutively recruited patients presenting with AECOPD who had a physician-diagnosis of COPD based on a history of cigarette smoking and a post-bronchodilator FEV1/VC <70%. Inclusion was also dependent on having a sputum cell count and differential and hemosiderin-stain available associated with the exacerbation. Exclusion criteria included known collagen vascular disease or active hemoptysis.

### Data collection

Sputum was either obtained spontaneously or was induced by inhalation of increasing concentrations of hypertonic saline at the time of exacerbation. Differential cell count and smoker’s inclusions within AMs were quantified after applying Wright’s stain [22, 23]. Prussian blue stain was used to detect hemosiderin in AMs, to allow for calculation of the hemosiderin index [23]. The hemosiderin index is defined as the percentage of AMs that contained hemosiderin granules observed after staining. Sputum IL-6 in cell-free supernatant was measured by ELISA (R&D Systems, Minneapolis, MN). Echocardiographic data, including right ventricular systolic pressures and left ventricular end-diastolic pressures, was collected from the electronic health records where available. The number of infective exacerbations in the previous two years was quantified, and was defined via chart review as physician diagnosed events that required an antibiotic for increase in sputum volume or color, or symptoms.

### Statistical analysis

Descriptive data are reported as means ± SD for parametric data or medians with IQR for non-parametric data. The relation between infective exacerbations (dependent variable) in the previous two-year period and the hemosiderin index (independent variable) was examined by simple regression analysis. A multivariate negative binomial regression model was used to determine the effect of hemosiderin index on infective exacerbations while adjusting for covariates. All analyses were performed using Dell Inc. Dell Statistica (Version 12) and R V.3.3.2 (R Foundation for Statistical Computing, Vienna, Austria).

## Results

**Fig. 1:**
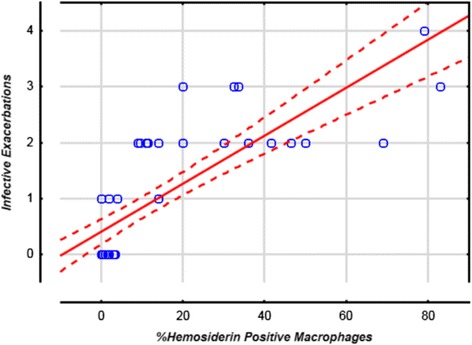
Previous Infective Exacerbations versus Hemosiderin Positive Macrophages (%). Linear regression with 95% Confidence Intervals

**Fig. 2:**
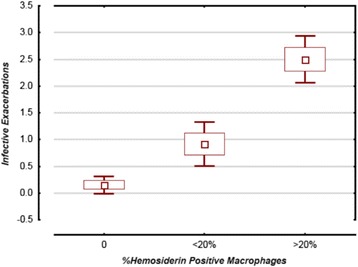
Previous Infective Exacerbations versus Hemosiderin Positive Macrophages (0%, <20%, and ≥20%). One-way ANOVA (95% Confidence Intervals)

**Fig. 3:**
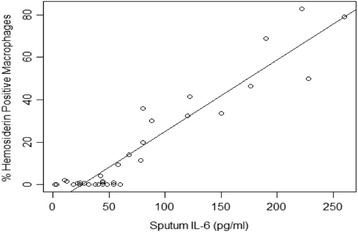
Hemosiderin Positive Macrophages (%) versus Sputum IL-6 (pg/ml). Linear regression

The patient characteristics are summarized in Table 1. The mean severity of airflow obstruction was moderate. The number of infective AECOPD within the previous two years are also summarized in Table 1, with approximately half of the subjects only having one episode. The results of the sputum total cell count and differential, as well as hemosiderin index, and IL-6 are summarized in Table 2. In terms of clinically relevant sputum phenotypes, most were categorized as pauci-granulocytic (27.8%) or neutrophilic bronchitis (24%). Sputum measures were available for all subjects, aside from IL-6 values, which were only available for 36. Using linear regression, there was a statistically significant correlation between hemosiderin index and the frequency of prior infective exacerbations (*r* = 0.79, *p* < 0.0001; Fig. 1). Using ANOVA, when subjects were categorized according to their hemosiderin positive macrophage levels (0%, <20%, or ≥20%), there was still a significant correlation with infective AECOPD (Fig. 2; *p* < 0.0001). Absolute neutrophils were not associated with hemosiderin index by ANOVA (*p* = 0.07). Those in the highest hemosiderin index group had significantly more frequent infective exacerbation than the other groups, with a mean of 2.5 (95%CI: 1.99-3.01). As over-dispersion was present (as indicated by the variance of infective exacerbations being higher than the mean), a negative binomial regression model was used. In this model, hemosiderin index predicted the number of exacerbations with a rate ratio of 1.04 (95% CI: 1.02–1.05) when adjusted for absolute neutrophil count, smoker’s inclusions (used as a surrogate for cigarette smoke exposure), and FEV_1_. Only hemosiderin index was a statistically significant predictor of infective AECOPD (*p* < 0.0001). The statistics are summarized in Table 3. Linear regression demonstrated a strong correlation between sputum IL-6 and hemosiderin index (Fig. 3, *r* = 0.95, *p* < 0001).

**Table 1:** Subject characteristics

Subject characteristics	Data
Number of Subjects	54
Average Age in Years	64.4 (±11.8)
Sex:	
• Number of Male • Number of Females	34 (63%) 20 (37%)
FEV_1_ % Predicted VC (L)	67.6% (±20.8) 3.37 (±0.92)
Active Smoking	46 (85%)
Cigarette Consumption:	
• Few • Moderate • Many	19 (41.3%) 17 (37.0%) 10 (21.7%)
Number of Prior Infective Exacerbations:	
• 0 • 1 • 2 • >2	29 (53.7%) 6 (11.1%) 14 (25.9%) 5 (9.3%)

Data are presented as mean ± SD or Number (%). *FEV*_*1*_ Forced expiratory volume in one second, *VC* Vital capacity

**Table 2:** Summary of sputum results

Sputum characteristics	Data
Total Cell Count × 10^6^/g	6.5 (2.5, 12.8)
Hemosiderin Index (%)	1.5 (0, 14)
Hemosiderin Category:	
• 0% • <20% • ≥20%	20 (37%) 22 (41%) 12 (22%)
IL-6 (pg/ml)	46.0 (24, 84)
Neutrophil (%)	65.6 (50, 79)
Absolute Neutrophils × 10^6^/g	3.7 (1.7, 9.5)
Eosinophil (%)	0.3 (0, 1.6)
Absolute Eosinophils × 10^6^/g	0.01 (0, 0.2)
Cellular Phenotype	
• Neutrophilic Bronchitis • Eosinophilic Bronchitis • Mixed Bronchitis • BUS • Paucigranulocytic	13 (24.1%) 7 (13.0%) 1 (1.8%) 18 (33.3%) 15 (27.8%)

Data are presented as median and IQR or Number (%)

Neutrophilic bronchitis = Total Cell Count (TCC) ≥10 × 10^6^/g and Sputum Neutrophils ≥65%; Eosinophilic bronchitis = Sputum Eosinophils ≥3%; Mixed Bronchitis = fulfilling criteria for both neutrophilic and eosinophilic bronchitis; Bronchitis of Undetermined Significance (BUS) = TCC ≥ 10 × 10^6^/g with Sputum Neutrophils <65% and Sputum Eosinophils <3% or TCC < 1010 × 10^6^/g and Sputum Neutrophils ≥65%; Paucigranulocytic = not meeting criteria for any of the above

**Table 3:** Negative binomial regression model

Coefficients	Estimate	z	*P*-value	95% CI	Rate ratio	95% CI
Intercept	−0.65	−1.53	0.13	−1.54–0.13	0.52	0.21–1.14
% Hemosiderin +	0.04	5.26	<0.0001	0.02–0.05	1.04	1.02–1.05
Absolute Neutrophils (×10^6^/g)	0.04	1.31	0.19	−0.02–0.09	1.04	0.98–1.09
Smoking Moderate	0.09	0.28	0.78	−0.59–0.76	1.10	0.56–2.13
Smoking Many	−0.95	−1.84	0.07	−1.99–0.02	0.39	0.14–1.02
FEV_1_ %Predicted	−0.01	−0.96	0.34	−0.03–0.01	0.99	0.97–1.01

## Discussion

Our results indicate a novel observation that the hemosiderin in AMs in sputum may predict the number of infective exacerbations in the preceding two years in patients with COPD, which may occur through an IL-6 dependent mechanism. For each 1% increase in hemosiderin positivity, there was an estimated 4% increase in the number of infective AECOPD, when adjusted for sputum neutrophils, smoking and FEV_1_. It is not clear if the presence of hemosiderin-laden AMs in these individuals occurs secondary to the underlying cause of AECOPD (e.g. infection), or if it represents a pre-existing risk factor for infective AECOPD. Nonetheless, the longevity of AMs suggests that this predictor is valid over multiple years, and thus it may hold value over other markers, such as IL-6, that may ebb and flow with exacerbations, and their related airway changes. Although there is evidence that increased respiratory iron content predisposes to infection, the mechanism remains to be clarified. Despite a small number of patients in our retrospective correlational study, these observations underscore the importance of a prospective study evaluating the hemosiderin index as a biomarker for frequent infective exacerbations of COPD.

**Fig. 4:**
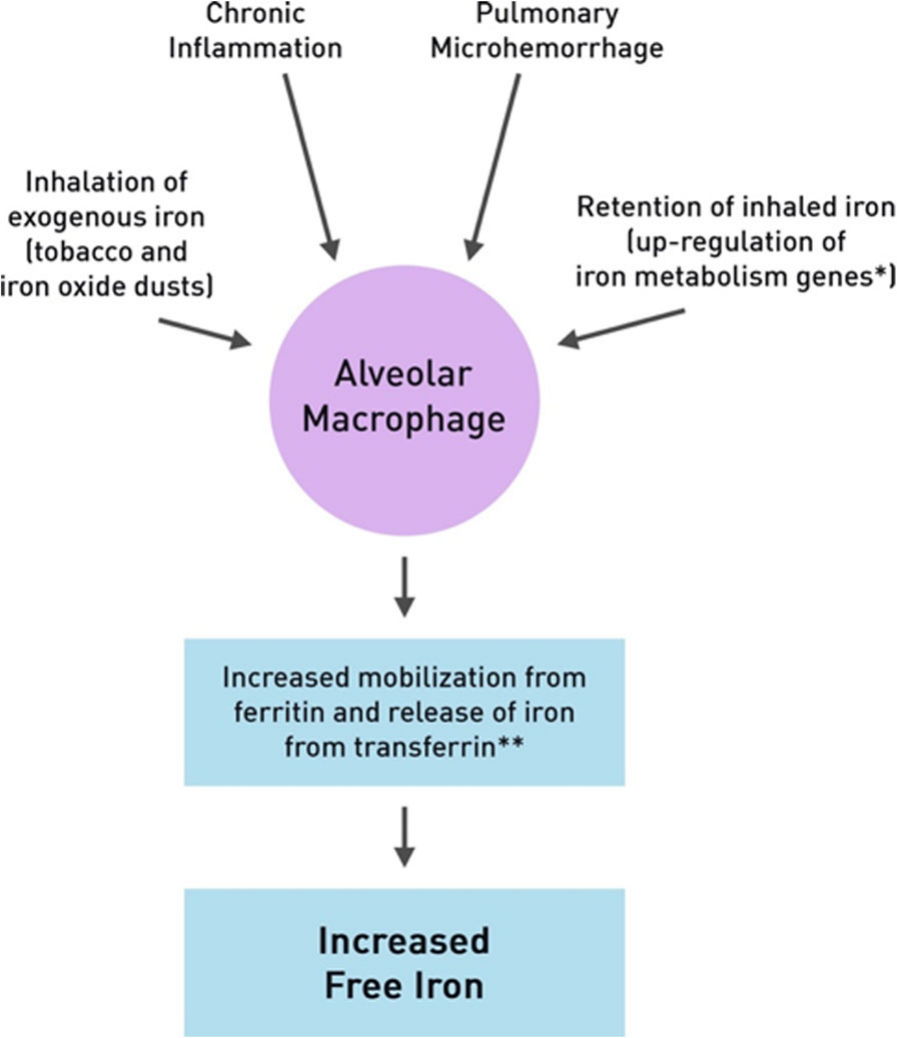
Mechanisms of Iron Uptake in Alveolar Macrophages. *Genes related to iron storage (Hemopexin and Haptoglobin), and export (Ceruloplasmin). **Mobilization by reducing agents and cleavage of transferrin

The mechanisms of iron uptake in AMs in COPD are multifactorial and are summarized in a schematic in Fig. 4. Evidence in mouse-models and in-vitro studies suggest that hepcidin, modulated by infection/inflammation (via cytokines such as IL-6), may lead to iron overload in the respiratory tract [24–26]. In our study, we found higher levels of sputum IL-6 in those with hemosiderin-laden AMs, which suggests that chronic inflammation may play a role in iron overload, causing local iron sequestration, and subsequent iron deprivation for pathogenic organisms [26]. It is plausible that IL-6 and its effects on iron metabolism may contribute to the frequent exacerbation phenotype, as studies have found that increased serum and BAL IL-6 levels are associated with poorer lung function, and frequent AECOPD [27, 28].

Chronic inflammation, whether it is related to “spillover” from airway inflammation inherent to COPD, genetically acquired polymorphisms, or recurrent infection, will cause the release of IL-6. This could lead to iron accumulation in the respiratory tract, which offers a favorable milieu for microbial growth and blunts macrophage function, and thus may contribute to infective AECOPD [29, 30]. On the other hand, it may be a respiratory infection in a predisposed individual that initiates the cycle. The development of infection activates inflammatory pathways including IL-6 (via NFκβ), but also directly activates hepcidin in AMs and airway epithelial cells via lipopolysaccharide, to cause local iron sequestration [25, 26]. Alternatively, iron overload may be occurring in susceptible individuals with a mutation in the iron metabolism pathway with a secondary insult. Regardless of the triggering event, it is plausible that hemosiderin in AMs is a complex biomarker encompassing factors including innate immunity, altered iron metabolism, and chronic inflammation. The contributory role of iron to exacerbations is consistent with the observations that all four proteins (α2-macroglobulin, haptoglobin, ceruloplasmin, and hemopexin) identified to discriminate between airway diseases and normal controls were related to iron metabolism [9].

This study has limitations. Being a retrospective design, a causal relation cannot be established from our results. A limited sample size prevented a robust multivariate analysis that would have adjusted for other variables, including sputum IL-6, which was only available for a subset of the subjects. Thus, we are unable to comment as to whether the prediction of infective exacerbation is mainly driven by IL-6 or hemosiderin positive AMs. In addition, echocardiographic data was limited to a minority of subjects, and was thus not used in the analysis. Extracellular iron content was not evaluated in these patients, although AMs constitute the major reservoir for excess iron in smokers [28]. Sputum was collected during an exacerbation, which may focus on an episode-specific change in iron content rather than during a steady state. Microbiology data was not available so the number of infective AECOPD was inferred from antibiotic use. Although active smoking status was not directly collected from subjects, smoker’s inclusions have been shown to be a reliable indicator of cigarette smoking [23]. While cigarette smoke may contribute to respiratory infections (though this correlation was not significant in our data), and contribute to hemosiderin-laden macrophages, our multivariate regression model accounted for smoker’s inclusions. In addition, hemosiderin and smoker’s inclusions in alveolar macrophages in sputum can be distinguished accurately based on appearance.

## Conclusions

Our study shows that a high hemosiderin index in sputum alveolar macrophages measured at the time of AECOPD may be related to the frequency of infective exacerbations in the preceding two years. This warrants a prospective study to determine if this relationship has predictive potential, as well as further research into the mechanisms of iron accumulation in the frequent infective exacerbation phenotype.

## Abbreviations

AECOPD: Acute exacerbation of chronic obstructive pulmonary disease
AM: Alveolar macrophage
BUS: Bronchitis of undetermined significance
COPD: Chronic obstructive pulmonary disease
FEV1: Forced expiratory volume in 1 s
IL-6: Interleukin-6
LVEDP: Left ventricular end-diastolic pressure
TCC: Total cell count
VC: Vital capacity
